# Neurotoxin Merging: A Strategy Deployed by the Venom of the Spider *Cupiennius salei* to Potentiate Toxicity on Insects

**DOI:** 10.3390/toxins12040250

**Published:** 2020-04-12

**Authors:** Benjamin Clémençon, Lucia Kuhn-Nentwig, Nicolas Langenegger, Lukas Kopp, Steve Peigneur, Jan Tytgat, Wolfgang Nentwig, Benjamin P. Lüscher

**Affiliations:** 1 Department of Nephrology and Hypertension, Inselspital, Bern University Hospital, Freiburgstrasse 15, 3010 Bern, Switzerland; 2 Institute of Ecology and Evolution, University of Bern, Baltzerstrasse 6, 3012 Bern, Switzerland; nicolas.langenegger@iee.unibe.ch (N.L.); lukas.kopp@gmail.com (L.K.); wolfgang.nentwig@iee.unibe.ch (W.N.); 3 Toxicology and Pharmacology, University of Leuven (KU Leuven), Campus Gasthuisberg, O & N 2, Herestraat 49, P.O. Box 922, 3000 Leuven, Belgium; steve.peigneur@kuleuven.be; 4 National Institutes of Health, Bethesda, MD 20892, USA; l_beni@hotmail.com

**Keywords:** neurotoxin merging, *Cupiennius salei*, venom, CsTx-13, microscale thermophoresis, bioassay

## Abstract

The venom of *Cupiennius salei* is composed of dozens of neurotoxins, with most of them supposed to act on ion channels. Some insecticidal monomeric neurotoxins contain an α-helical part besides their inhibitor cystine knot (ICK) motif (type 1). Other neurotoxins have, besides the ICK motif, an α-helical part of an open loop, resulting in a heterodimeric structure (type 2). Due to their low toxicity, it is difficult to understand the existence of type 2 peptides. Here, we show with the voltage clamp technique in oocytes of *Xenopus laevis* that a combined application of structural type 1 and type 2 neurotoxins has a much more pronounced cytolytic effect than each of the toxins alone. In biotests with *Drosophila melanogaster*, the combined effect of both neurotoxins was enhanced by 2 to 3 log units when compared to the components alone. Electrophysiological measurements of a type 2 peptide at 18 ion channel types, expressed in *Xenopus laevis* oocytes, showed no effect. Microscale thermophoresis data indicate a monomeric/heterodimeric peptide complex formation, thus a direct interaction between type 1 and type 2 peptides, leading to cell death. In conclusion, peptide mergers between both neurotoxins are the main cause for the high cytolytic activity of *Cupiennius salei* venom.

## 1. Introduction

With more than 48,000 species of terrestrial arthropods, spiders are the most diverse group after insects [1] and this success is often explained by their unique combination of silk and venom glands [2]. Spider venom is a rich source of bioactive substances, which are of great interest as a starting base for the development of new insecticides [3] and human therapeutics [4]. Venoms are in general composed of ions, small molecular mass compounds, neurotoxins, enzymes, and other proteins [5]. This complex mixture enables spiders to defend themselves against predators and to subdue prey. The immobilization of prey results from a complex cascade of substances, described as dual prey-inactivation strategy, in which simultaneously 1) enzymes and proteins interact with the regulation of important metabolic pathways in an unspecific manner, and 2) small molecular mass compounds and neurotoxins act highly specifically on their targets, different ion channels in muscles, and nervous tissue. In addition, synergisms between different neurotoxins and with cytolytic peptides, ions and small molecular mass compounds contribute considerably to the venom toxicity [6].

Mature neurotoxins are synthesized as inactive precursors, which are composed of a signal peptide, usually a propeptide and the neurotoxin. In a first step, the signal peptide is removed by the activity of a signal peptidase. After cutting the propeptide at the C-terminal-processing quadruplet motif (PQM) by the PQM protease, the neurotoxin undergoes further processing, summarized as post-translational modifications, such as disulfide bridge formation or C-terminal amidation [7].

**Figure 1:**
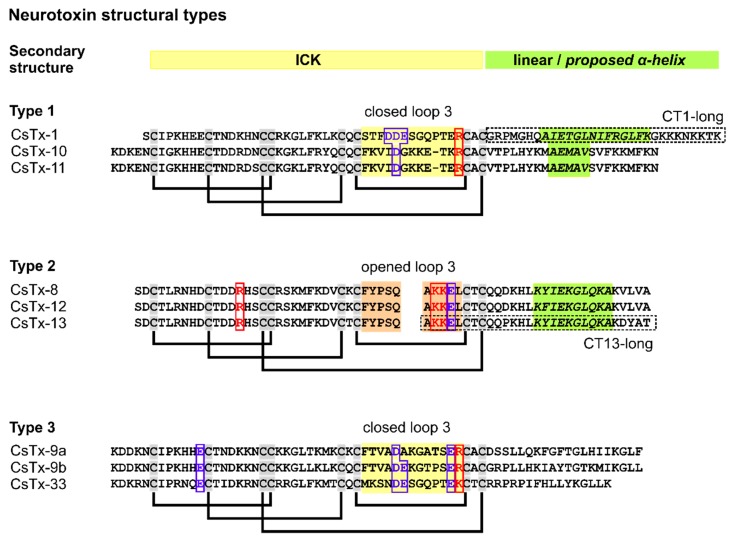
Sequence comparison and disulfide bridge arrangement of different neurotoxin structures from *Cupiennius salei*. Proposed C-terminal α-helical structures are shaded in light green and amino acid residues involved in forming the main part of loop 3 of the ICK structure are shaded in yellow/light brown. The possible docking region of heterodimeric neurotoxins and the proposed corresponding docking region of monomeric neurotoxins are boxed according to their charge. The involved cationic amino acid residues are in red and anionic amino acid residues are colored in blue. CT1-long and CT13-long are indicated by a box. Cysteines are highlighted in gray.

In the venom of *Cupiennius salei*, 76% of all cysteine containing neurotoxin-like transcripts exhibit, besides the N-terminally ICK motif, C-terminally an α-helical motif [6]. They are composed of three disulfide bridges as C1-C4, C2-C5, and C3-C8 and an additional fourth disulfide bridge C6-C7 (Figure 1). This first structural type, as represented by CsTx-1, the main active neurotoxin in the venom, accounts for, together with the structurally similar CsTx-10 and CsTx-11, 28% of all neurotoxins in the venom. The second structural type possesses, besides a two-domain architecture, a further post-translational modification in which the loop between the disulfide bridge C6-C7 is post-translationally opened by a specific protease. This protease recognizes a PQM and an inverted PQM motif within this loop, which results in a heterodimeric peptide as reported for CsTx-13 [6,8,9]. These post-translationally modified neurotoxins (CsTx-8, 12, and 13) together represent 48% of all neurotoxins, but they are overall 49 times less insecticidal than CsTx-1. On the other hand, such two-chain peptides are able to enhance the insecticidal activity of CsTx-1 and CsTx-9 even in very low concentrations [9]. A third structural type exhibits only the ICK motif and no C-terminal α-helix, as shown by CsTx-9 (7%) [6], resulting in a slightly higher insecticidal effect than the second structural type (Figure 1).

Here, we show that remarkable interactions between structural type 1 (or 3) and type 2 toxins, described as neurotoxin merging, lead to a strong increase of venom toxicity. This result offers new access to the high diversity of venom compounds.

## 2. Results

### 2.1. Cytolytic Effects of CsTx-1 and CsTx-13 or CsTx-9 in Xenopus Oocytes Plasma Membranes

**Figure 2:**
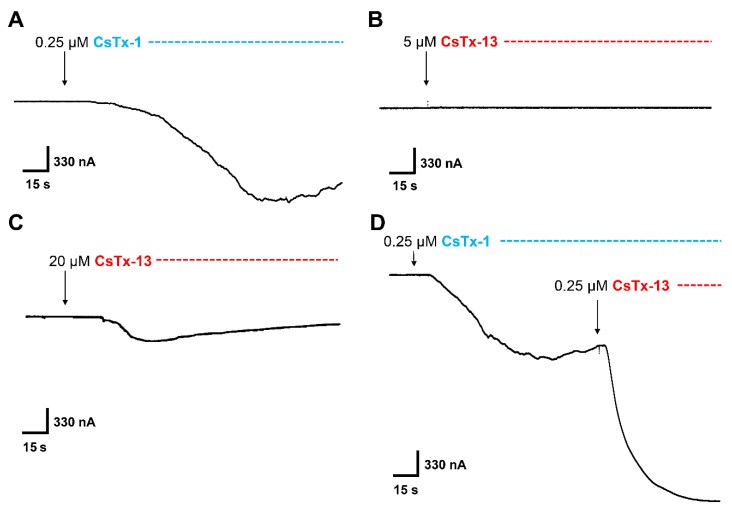
Effect of CsTx-1, CsTx-13, and the combination of both in *Xenopus laevis* oocytes. The membrane potential of denuded oocytes was adjusted to -40 mV. (**A**) Exposure of CsTx-1 (blue) at the 0.25 µM concentration results in an inward current amounting to several μA gradually developing. (**B**) CsTx-13 (red) has no effect on the resting current of oocytes up to the 5 µM concentration. However, (**C**) CsTx-13 induces an inward current at the 20 µM concentration, comparable to CsTx-1 at the 0.25 µM concentration. (**D**) The CsTx-1-induced current is amplified 1.8-fold after the application of CsTx-13 at an equal molar concentration (0.25 µM).

With the two-electrode voltage clamp technique, we found for *Xenopus laevis* oocytes’ membranes remarkable cytolytic effects of CsTx-1 and CsTx-13. CsTx-1 (0.25 µM) induced an inward current in the range of 0.5 to 8 µA on the clamped membrane potential at −40 mV (Figure 2A). This increase of the current is due to the cytolytic effect of the C-terminal α-helix, which breaks the membrane resistance and induces an increase of the ion flow through the membrane by affecting the outer leaflet curvature and/or pore formation [10]. CsTx-13 (0.25 µM), which has a shorter C-terminal α-helix than CsTx-1 (Figure 1), exhibited no cytolytic activity up to 5 µM (Figure 2B). Nevertheless, in an 80-fold higher concentration than CsTx-1, CsTx-13 (20 µM) showed a cytolytic effect (Figure 2C). Interestingly, pre-incubation of the oocyte with CsTx-1 (0.25 µM), subsequently followed by the addition of CsTx-13 (0.25 µM), showed a 1.8-fold increase of the inward current (Figure 2D). We conclude from these results that a specific interaction between both peptides occurred because CsTx-13 alone exhibited cytolytic effects only in much higher concentrations.

**Figure 3:**
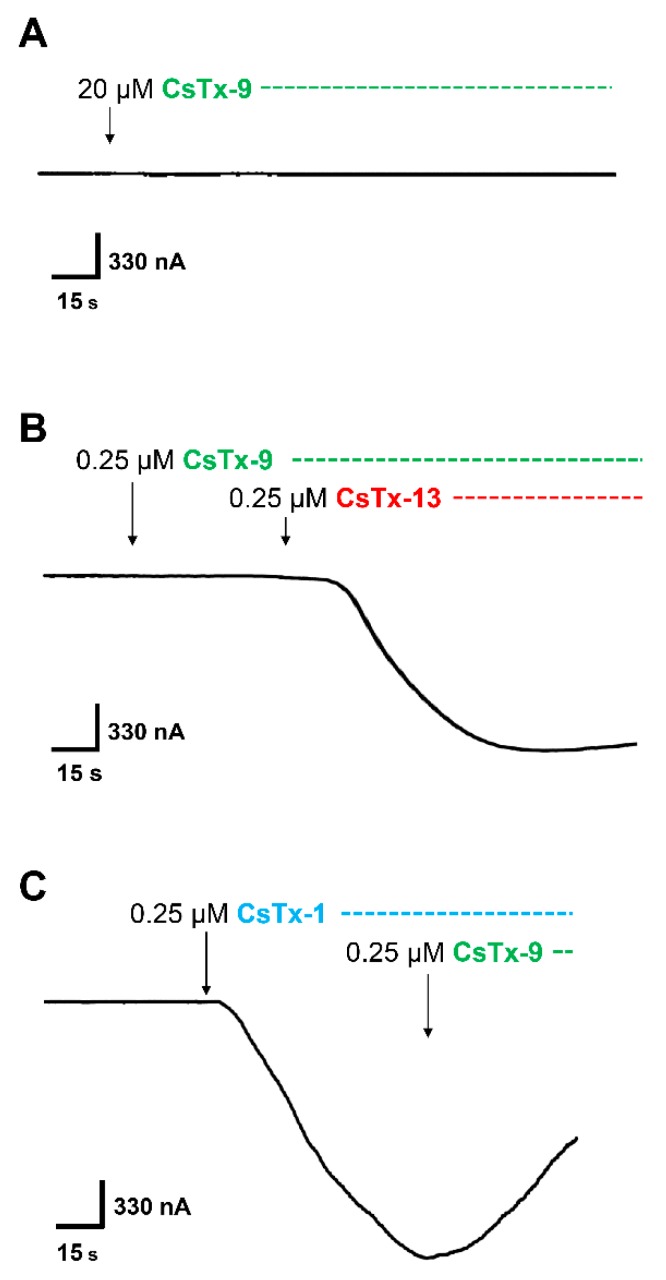
Effect of CsTx-9, and in combination with CsTx-13 or CsTx-1 in *Xenopus laevis* oocytes. (**A**) CsTx-9 (green) did not induce a current up to the 20 µM concentration. (**B**) A serial application of CsTx-9 (0.25 µM) followed by an application of CsTx-13 (red) in an equal molar ratio (0.25 µM) resulted in an inward current comparable in size to the current amplitude induced by CsTx-1 and CsTx-13 at an equal molar concentration (Figure 2D). (**C**) CsTx-9 did not affect the CsTx-1 (blue)-induced current in a serial application after reaching the plateau phase of the CsTx-1-induced current, indicating that no interaction occurred between CsTx-1 and CsTx-9.

Incubation of the oocyte with CsTx-9 alone, which possess only the ICK motif without a C-terminal α-helix (Figure 1), showed no cytolytic activity up to 20 µM (Figure 3A). Surprisingly, incubation of the oocyte with CsTx-9 (0.25 µM), subsequently followed by the addition of CsTx-13 (0.25 µM), resulted in strong cytolytic activity (Figure 3B), comparable to the one observed with the combination of CsTx-1 and CsTx-13. An enhancement of the CsTx-1 (0.25 µM)-induced current by CsTx-9 (0.25 µM) was not observed (Figure 2A and Figure 3C).

### 2.2. Insecticidal Activity

**Figure 4:**
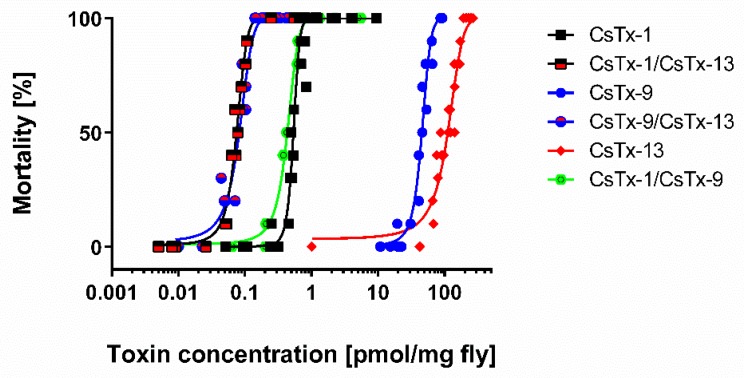
Insecticidal activity of the heterodimeric CsTx-13 alone, and in combination with the monomeric neurotoxins CsTx-1 and CsTx-9 on *Drosophila* flies. For the bioassays, different concentrations of CsTx-1, CsTx-9, and CsTx-13 alone, or in combinations of CsTx-1 and CsTx-9, CsTx-1 and CsTx-13, and CsTx-9 and CsTx-13 (1:1 molar ratio), were injected in 0.1 M ammonium acetate, pH = 6.1 (injected volume 50 nL). Each data point represents 10 injected flies. Calculations of the lethal dose (LD_50_), where 50% of the injected flies died of intoxication after 24 h, were done with GraphPad PRISM vers. 6.07. A negative control was done with injection of 50 nL of the abovementioned buffer alone into the flies.

In bioassays with *Drosophila* flies, we demonstrated a comparable peptide–peptide interaction of CsTx-13 when co-injected with CsTx-1 or CsTx-9. First, we injected different concentrations of CsTx-1, CsTx-9, and CsTx-13 alone into the flies. The main neurotoxin CsTx-1 (LD_50_ 0.535 pmol/mg fly; 95% confidence interval 0.515 to 0.555) was found to be about 86 times more toxic than CsTx-9 (LD_50_ 45.54 pmol/mg fly; 95% confidence interval 43.30 to 47.78), and about 208 times more toxic than the dimeric neurotoxin CsTx-13 (LD_50_ 111.2 pmol/mg fly; 95% confidence interval 105.5 to 116.9). Second, co-injection of the monomeric neurotoxins CsTx-1 and CsTx-9, in a 1:1 molar ratio, resulted in a 1.2-fold increase of toxicity (LD_50_ 0.432 pmol/mg fly; 95% confidence interval 0.417 to 0.447) when compared with CsTx-1 alone. Coinjection of the dimeric neurotoxin CsTx-13 with the monomeric neurotoxin CsTx-1 in equal molar ratios (LD_50_ 0.075 pmol/mg fly; 95% confidence interval 0.072 to 0.078) showed a 1487-fold increase of toxicity, when compared with CsTx-13 alone. Surprisingly, co-injection of CsTx-13 with CsTx-9 in equal molar ratios (LD_50_ 0.082 pmol/mg fly; 95% confidence interval 0.077 to 0.087) also showed a 1357-fold increase of toxicity, when compared with CsTx-13 alone (Figure 4).

### 2.3. Effects of CsTx-13 on Ion Channels

We investigated the possible effect of CsTx-13 (500 nM) on 18 different ion channels expressed in oocytes: K_V_1.1, K_V_1.2, K_V_1.3, K_V_1.4, K_V_1.5, K_V_1.6, K_V_2.1, K_V_3.1, K_V_4.2, K_V_4.3, Shaker IR, hERG, Na_V_1.2, Na_V_1.4, Na_V_1.5, Na_V_1.6, DmNa_V_1, and Ca_V_3.3. No significant activity of CsTx-13 on these ion channels was observed (Supplementary Figure S1).

### 2.4. Molecular Neurotoxin Interactions Revealed by Microscale Thermophoresis (MST)

**Figure 5:**
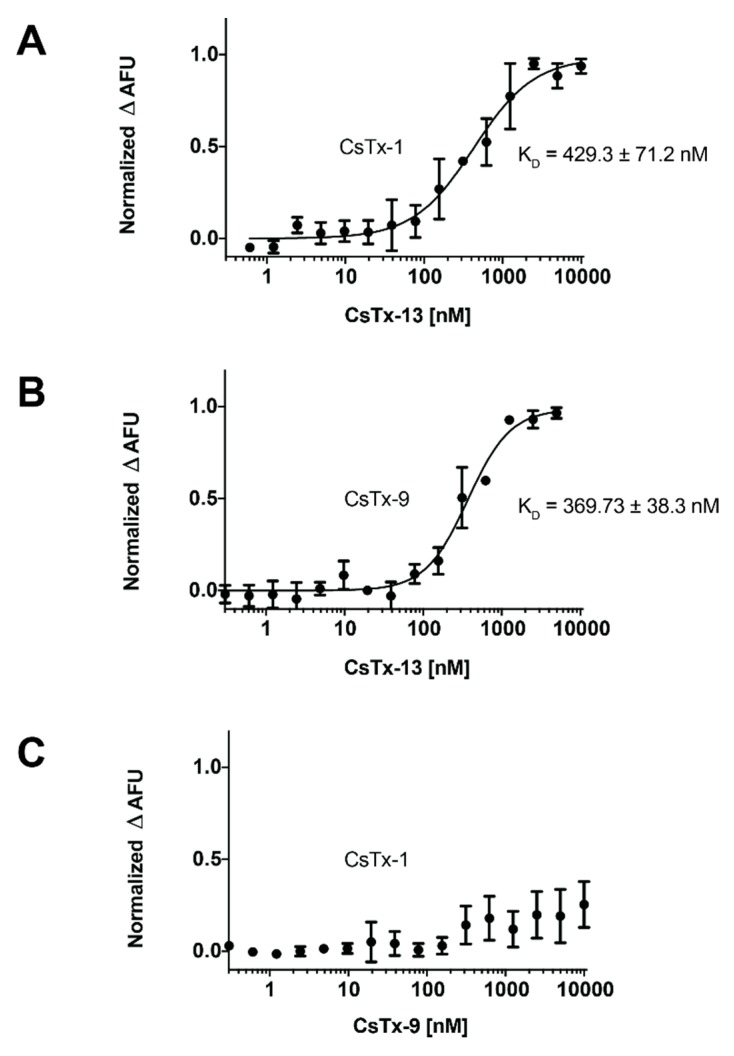
Peptide–peptide interaction of CsTx-1/9 with CsTx-13 using MST. (**A**) Titration using CsTx-13 neurotoxin to CsTx-1 was performed. High-affinity binding of CsTx-13 to CsTx-1 was measured with K_D_ equal to around 430 nM (**B**) Similar K_D_ was observed for CsTx-9 at 370 nM. (**C**) No binding interaction occurred between CsTx-1 and CsTx-9. In all cases, data were standardized to bound fractions.

We used the MST technique to verify and characterize the hypothesized monomeric/heterodimeric peptide complex formation at the molecular level of CsTx-13 with CsTx-1 or CsTx-9 (Figure 5). Purified and N-terminally labelled CsTx-1 or CsTx-9 were titrated with different concentrations of CsTx-13 (Figure 5A,B). Highly specific bindings between CsTx-13 with CsTx-1, or CsTx-13 with CsTx-9, respectively, at nanomolar concentrations were measured. Indeed, K_D_ of 430 and 370 nM were obtained for CsTx-1 and CsTx-9, respectively. We further confirmed that no peptide–peptide merging occurred between CsTx-1 and CsTx-9 (Figure 5C), which is comparable to the results obtained in the oocyte experiments, as well as in the *Drosophila* bioassays. However, although direct interactions between CsTx-13 and CsTx-1/9 were confirmed, the responsible peptide domains involved in this interaction remain unknown.

### 2.5. Influence of C-Terminal α-Helices on the Cytolytic Activity of CsTx-1 and CsTx-13

To estimate the impact of the C-terminal α-helices of CsTx-1 and CsTx-13 on the above identified cytolytic activity of the peptide–peptide complex, we synthesized the C-terminal sequence part of both neurotoxins and named them, corresponding to the original toxins, CT1-long and CT13-long (Figure 1). The predicted secondary structure of CT13-long was verified by circular dichroism spectroscopy (CD) (Table 1). Recording the CD spectra of CT13-long in the presence of PBS buffer, it was found that the peptide mainly adopts the proportions of a β-sheet, a β-turn, and an unordered structure. However, in the presence of TFE, a pronounced spectral change was observed in which the α-helical structure content increased from 1% to 74 %, with a concurrent decrease of the β-sheet, β-turn, and the unordered structure contents, corresponding to CT1-long.

**Figure 6:**
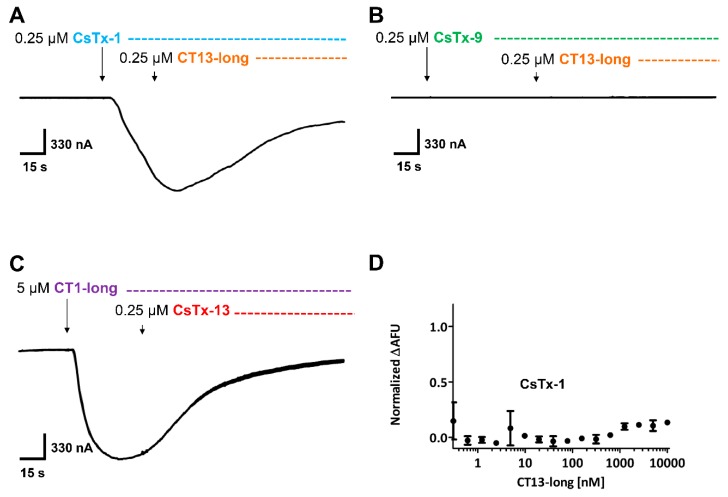
Identification of interacting protein domains in *Xenopus laevis* oocyte membranes. (**A**) CT13-long (orange) had no effect on the CsTx-1 (blue)-induced current. (**B**) CT13-long (orange) was not able to induce a current in combination with CsTx-9 (green). (**C**) CT1-long (purple)-induced current could not be amplified using CsTx-13 (red). (**D**) No protein–protein interaction could be observed between CsTx-1 and CT13-long using MST. Data were standardized to the Δ fluorescence shift between bound to unbound fractions of CsTx-1 and CT13-long.

CT1-long induced a current in oocytes only in an 80-fold higher concentration (20 µM) (Supplementary Figure S2A) when compared with CsTx-1 (0.25 µM). Moreover, CT13-long exhibited a first current only at a concentration of 400 µM, which is a 20-fold higher concentration than that determined for the heterodimer CsTx-13 (20 µM) alone (Supplementary Figure S2B). Incubation of oocytes either with CsTx-1 and CT13-long (both 0.25 µM) (Figure 6A), or CsTx-9 and CT13-long (both 0.25 µM) (Figure 6B) did not exhibit any cytolytic activity.

The inward current of CsTx-1 alone was not increased by CT13-long. Furthermore, the inward current induced by CT1-long (5 µM) alone was not enhanced by CsTx-13 (0.25 µM) (Figure 6C). Comparably, no molecular interactions between CT13-long and CsTx-1 were identified by the MST approach (Figure 6D), thus the C-terminal α-helix of CsTx-13 did not interact with the monomeric peptides. Vice versa, the 20-fold higher active C-terminal α-helix of CsTx-1 alone was not able to increase the cytolytic activity of CsTx-13.This indicates that the cytolytic activity of the proposed peptide–peptide complex depends not only on the activity of their C-terminal α-helices. In fact, the dramatic cytolytic increase of the combination of a heterodimeric with a monomeric neurotoxin at very low concentrations first requires the merging of both peptides, followed by structural changes.

### 2.6. Molecular Modelling of CsTx-1, CsTx-9, and CsTx-13

**Figure 7:**
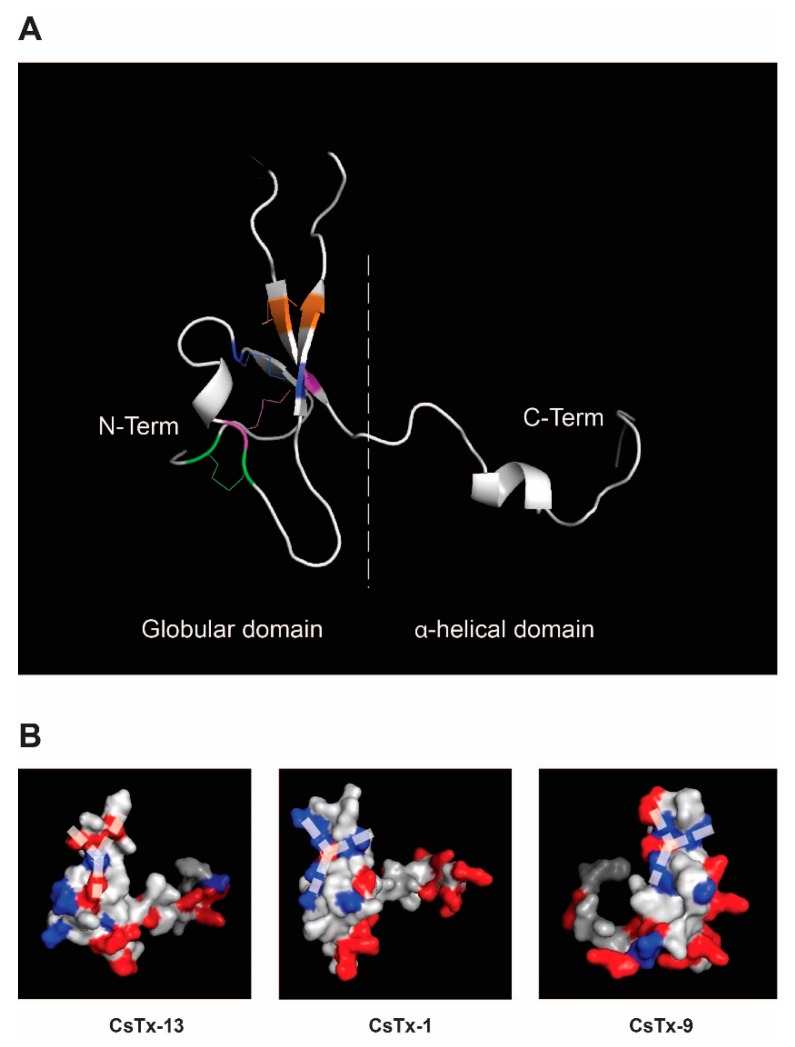
Homology model of CsTx-1, CsTx-9, and CsTx13 based on the NMR structure of purotoxin-2. (**A**) The predicted 3D structure of CsTx-13 was visualized in a cartoon representation and allows the relative position of the secondary structures within the model to be distinguished. The visualization showed two domains: (i) An α-helical domain at the C-terminal end composed of an α-helix (Y56-G60) responsible for the cytolytic activity of the neurotoxin, and (ii) a globular domain at the N-terminal part exhibiting the ICK motif composed of 3 β-sheets linked with 4 disulfide bonds colored in green (C3-C18), blue (C10-C27), purple (C17-C48), and orange (C29-C48). (**B**) The surface representation allows evidence of the spatial localization of all positive (red) and negative (blue) amino acid residues within the overall predicted structures and highlights “Y” shape electrostatic interaction domains responsible for neurotoxin merging, which is composed of loop 3 and part of the ICK of CsTx-13, CsTx-1 and CsTx-9, respectively.

With the exclusion of the C-terminal α-helix of CsTx-13 alone as a driving force for the observed strong cytolytic effects between CsTx-13 and CsTx-1/9, we focused on the ICK motif of both peptide types. Especially, the open loop 3 between Cys6 and Cys7 of the structural type 2 neurotoxins (Figure 1) drew our attention. This open loop is the result of a post-translational modification as described earlier [8]. Homology modelling of CsTx-13 based on the recently solved NMR structure of purotoxin-2 [13] and Phyre2 analysis [14] revealed that the purotoxin-2 structure was the best template based on sequence alignment analysis. Purotoxin-2 shared 56.5% with CsTx-13, 28% with CsTx-9, and 35% sequence identity with CsTx-1, covering 80% to 90% of the overall protein (Supplementary Figure S3). Predictive structures were generated using Modeller v9.19 program [15]. Mature CsTx-13 is composed of two structural motifs: The N-terminal ICK motif and a C-terminal α-helix. Three disulfide bonds between C3-C18, C10-C27, and C17-C42 represent the ICK motif and an additional bond between C29-C40 forms the open loop (Figure 7A). The C-terminal end is composed of a long tail of 21 amino acid residues with an α-helix structure from K49 to A58. The same approach was used to generate the predictive structures of CsTx-1 and CsTx-9. Surface modelling allowed the identification of the specifically exposed region of the corresponding peptide loop (S29 to R41 for CsTx-1 and F33 to R45 for CsTx-9). This representation showed a specific Y-shape motif of the charged amino acids inside the loop domain. CsTx-13 exhibits two positive charges in the center of the Y-motif surrounded by three negatively charged amino acids. Interestingly, the same structure was observed within the loop domain of CsTx-1 and CsTx-9 (Figure 7B) but with the opposite charge composition. This molecular evidence could explain our experimental data and suggests an electrostatic binding interaction between CsTx-13 and CsTx-1/9 involving at least four charged amino acids (Figure 1 and Figure 7B). This suggests that the regions of the ICK motifs of CsTx-1 and CsTx-9, especially the loop between Cys6 and Cys7, may bind to the open loop in the N-terminal part of CsTx-13 and influence its overall structure. This peptide merging strongly enhances the cytolytic activity, thus the toxicity of the spider venom.

## 3. Discussion

### 3.1. Different Structural Types of Neurotoxins

The evolution of neurotoxins has resulted in a tremendous variety of structures in the venoms of spiders [5]. While the classical spider neurotoxins consist of one peptide chain and one motif, Vassilevski and coworkers flagged other structures and created the umbrella term of modular toxins to differentiate between one motif- and two motif-containing neurotoxins [13,16]. Our structural types 1 (CsTx-1) and 2 (CsTx-13) refer to the modular neurotoxins exhibiting an N-terminal ICK motif and a C-terminal α-helix, as reported previously [9,10]. Additionally, the structural type 2 neurotoxins CsTx-8, CsTx-12, and CsTx-13 [6] are, due to the open loop, characterized by their heterodimeric structure, which seems to be the source for the observed remarkable enhancement of the insecticidal activity of modular and simple neurotoxins [9]. Overall, we could imagine that modular toxins are much more common in spider venoms than previously thought, because they provide a much higher toxicity.

### 3.2. Effects of C-Terminal α-Helical Structures of Modular Toxins in Oocytes

The enhancement effect of CsTx-13 on the insecticidal activity of CsTx-1 or 9, published 15 years ago [9], was re-analyzed by investigating the effects of these neurotoxins alone and in combination in *Xenopus laevis* oocytes. Interestingly, the combination of CsTx-13 with CsTx-1 or CsTx-13 with CsTx-9, in a 1:1 molar ratio (0.25 µM), resulted in a two-fold increase of the cytolytic activity when compared with the current, which was obtained by CsTx-1 (0.25 µM) alone. The targets of these interactions are neutral membranes and not ion channels, which was confirmed by our negative results when testing a high diversity of ion channels with CsTx-13, assuming that these results can be generalized.

**Figure 8:**
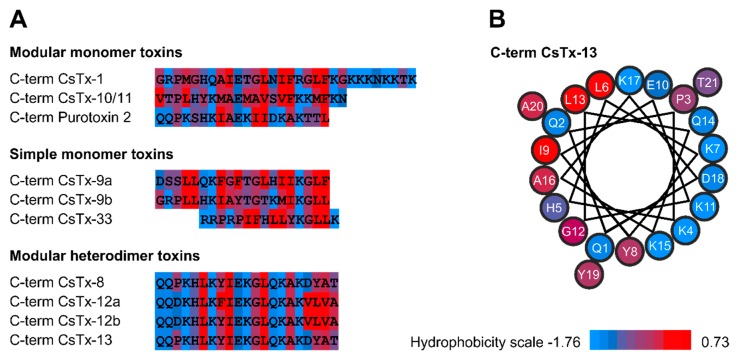
Hydrophobicity plot of C-terminal linear and α-helical structures of neurotoxins. (**A**) The consensus hydrophobicity scale of Eisenberg et al. [18] was used to characterize the hydrophobicity of each amino acid residue. (**B**) α-helical wheel projection of the C-terminus of CsTx-13 exhibiting distinct hydrophobic and polar regions.

Meanwhile, it is well documented that the C-terminal linear part of CsTx-1 [10], and the N-terminal linear part of the spiderine (OtTx1a) [16,17] alone are able to adopt an α-helical structure in the presence of membranes or membrane-mimicking agents, resulting in cytolytic activities. As part of the modular toxins, such as α-helical structures, are supposed to link the neurotoxins to membranes and to act as an anchor, such as in the case of CsTx-1, which is caused by its unusually highly cationic charged C-terminus [10,13,17]. Therefore, we focused on the C-terminal α-helix of CsTx-13 (CT13-long) as the possible driving force for the enhancement described above. CT13-long is able to adopt an α-helical structure in a membrane-mimicking environment, comparable to CT1-long [10] and PT2-C [13]. However, CT13-long (400 µM) needed a 20-fold higher concentration than CsTx-1 (20 µM) to induce a leakage current on oocytes. This result is consistent with the findings that the C-terminal part of purotoxin 2, PT2-C, shows up to a concentration of 200 µM no antimicrobial activity against different bacteria [13], although PT2-C and CT13-long exhibit an amphiphilic structure and a high sequence identity (Figure 8).

### 3.3. Mode of Action

**Figure 9:**
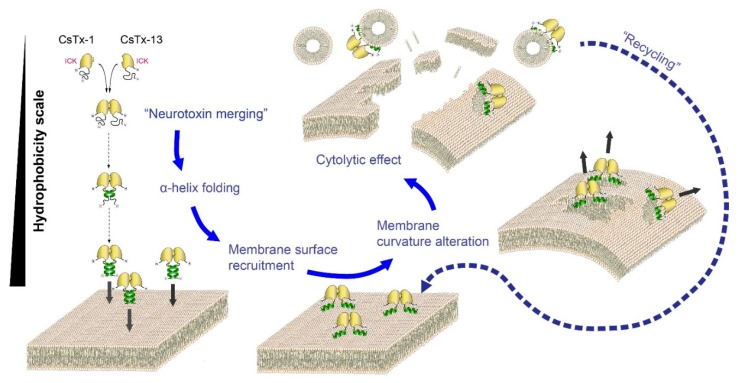
Proposed mode of action of neurotoxin merging. Our model presents neurotoxins as water-soluble molecules with non-structured C-terminal ends. CsTx-13 is able to bind to CsTx1/9 via their N-terminal globular domains, which include loop 3 as well as parts of the ICK motifs. These merged neurotoxins possess spatially closer C-terminal ends, which mimic a hydrophobic environment and may subsequently lead to α-helical formation in the presence of membranes. This conformational change induces an increase of the hydrophobicity pattern of the merged peptides. Insertion of the hydrophobic areas of the α-helices into the membrane induces an alteration of the membrane curvature, leading to a thinning out of the outer leaflet lipids and the formation of lipid/merged neurotoxin micelles, which finally will induce the disruption of the membrane lipid bilayer observed as a cytolytic effect in vitro. A “recycling” process for the peptide merger is hypothesized.

First, we propose membrane binding of the merged peptides by amplified hydrophobic interactions with a shared hydrophobic patch, generated by both C-terminal α-helices of CsTx-13 and CsTx-1. This may result in a thinning of the outer membrane leaflet, e.g., in an altered membrane curvature. Second, after reaching a critical concentration of merged peptides on/in the outer leaflet of the membrane, membrane leakage may occur through an unknown mechanism, finally leading to cell death (Figure 9).

For the cytolytic activity of α-helical antimicrobial peptides, three different mechanisms have been proposed: The barrel stave pore mechanism, toroidal wormhole pore formation, and carpet-like mechanism [19]. Pore formation, however, needs α-helices spanning through the membrane, which is around two times thicker than the lengths of the α-helices of CsTx-1 and CsTx-13. Additionally, a specific lipid composition of the membrane is required to become penetrable by an α-helix, as shown for the highly cationic cupiennins, α-helical antimicrobial peptides, identified in the venom of *Cupiennius salei* [20]. The LD_50_ values (bioassays in *Drosophila* flies) of these cupiennins range between 4.7 and 7.9 pmol/mg fly [21] and are, by a factor of 100, less insecticidal than the peptide mergers CsTx-13/1 and CsTx-13/9. Taking these results into account, the herein proposed hydrophobic mechanism represents a fourth mode of interaction with membranes that leads to their destruction (Figure 9). A possible recycling of the peptide mergers CsTx-13/Cstx-1 and CsTx-13 / CsTx-9 during the membrane permeabilization process is hypothesized as a possible explanation for the low LD_50_ values of the peptide mergers.

So far, most α-helical and cytolytic-acting peptides in spider venoms are characterized by a high cationic charge and an amphipathic character [22,23]. Membrane attraction is supposed through electrostatic interactions between negatively charged membrane components (phospholipid head groups, glycoproteins, lipid rafts), and the positively charged lysines and arginines of the cytolytic peptides, resulting in membrane damage [21,22].

The hypothesis of the mainly hydrophobic interactions of the peptide merger with membranes as the driving force is supported by the peptide merger of CsTx-13 and CsTx-9, which is characterized by only one C-terminal α-helix and the hydrophobic unstructured C-terminus of CsTx-9. NMR spectroscopy of PT2 in water shows a highly rigid ICK knottin core and more flexible linear N-terminal and C-terminal structures. However, in the presence of membrane-mimicking dodecylphosphocholine micelles, the C-terminus adopts an α-helical structure with lower mobility [13]. Comparably, in the presence of the oocyte membrane, the peptide complex between CsTx-13 and CsTx-1/9 may be characterized by rigid ICK knottin cores and a lower C-terminal mobility, which induces a greater hydrophobic patch, either formed by two C-terminal α-helical structures or by one α-helical structure and an unstructured hydrophobic C-terminus, respectively.

### 3.4. Evolutionary Aspects

Among spiders, the “retrotibial apophysis clade” (RTA clade or family group) is considered to represent one of the highest evolved spider groups [24]. Modular toxins composed of two ICK motifs in cheiracanthiids [25] or two α-helical motifs in zodariids [26] are reported. Within ctenids, lycosids, oxyopids [13,16], and *Cupiennius* [6], modular toxins composed first of an ICK-motif followed by an α-helical motif, or vice versa, have been identified. Moreover, cytolytic highly cationic α-helical peptides, acting on a diversity of negatively charged membranes, are so far only known from lycosids, zodariids, oxyopids [22], and *Cupiennius* [21,27]. The joint occurrence of cytolytic peptides and modular toxins in this modern family group points to the solution of a potential problem when relying only on highly specific neurotoxin interactions with ion channels and receptors. Here, we can only speculate on the nature of this problem, which may perhaps be in the field of resistance development by prey types against some neurotoxins.

## 4. Conclusions

With the herein described peptide merging between the heterodimeric CsTx-13 and simple or modular neurotoxins, we identified a further mode of action for spider venom, targeting neutral membranes and resulting in increased insecticidal activity by 2 to 3 log units. This is a further step away from a specific toxin/receptor/ion channel interaction only, as found in mygalomorph spider venoms, to the much broader-acting venom we described here for spiders of the RTA clade.

## 5. Materials and Methods

### 5.1. Spider Maintenance, Venom Collection, and Neurotoxin Purification

Spiders were laboratory bred and venom was obtained by electrical stimulation as previously described [28]. Purification of CsTx-1, CsTx-13, and CsTx-9 was done by a combination of FPLC and RP-HPLC as reported earlier [9,28,29]. CT1-long, and CT13-long were synthesized by GeneCust (Laboratoire de Biotechnologie du Luxembourg S.A.). Peptide concentrations were determined in triplicate by amino acid analysis [9].

### 5.2. Circular Dichroism (CD) Measurements

CD measurements of CT13-long were performed and analyzed as described for CT1-long [10,11,12,30]. Briefly, CT13-long (40 μM) was dissolved in a buffer composed of 5 mM Na_2_HPO_4_/NaH_2_PO_4_, pH = 7.2, and 150 mM NaF or in the same buffer containing 50% (v/v) 2,2,2-trifluoroethanol (TFE) and measurements were performed with a Jasco J-175 spectropolarimeter in a Suprasil R110-QS 0.1 cm quartz cell (range of 178–260 nm, 20 °C).

### 5.3. Xenopus laevis Oocytes Preparation

Frog surgery was done as described in [31] with the following changes in the protocol: The female frogs were laid on wet tissues instead a bed of ice to avoid frog skin irritation. The sterile filtered Barth’s medium contained gentamicin (50 μg /mL) as antibiotics. Peeling of the oocytes was carried out as described in [31]. In these experiments, the oocytes were exposed for 20 min at 34 °C instead of 36 °C, as this procedure is less harmful for the oocytes. Oocytes were then conveniently freed from the surrounding layers by rolling them in a non-coated plastic culture dish, which simplified the removal of the follicular layer. Oocytes were stored for at least 120 min in sterile filtered Barth’s medium at 18 °C to allow recovery from the procedure. Oocytes with a membrane potential of 0.8–1.2 MΩ were used for experiments.

### 5.4. Two Electrode Voltage Clamp Experiments

The two-electrode voltage-clamp method was used to measure currents elucidated by the neurotoxins in *Xenopus laevis* oocytes according to Sigel [31] with the following changes: Oocytes were kept at a holding potential of −40mV and 10 μL of toxin were applied directly to the bath (volume 200 μL). Currents were measured with an OC-725C amplifier (Warner Instruments, Hamden, CT, USA), low-pass filtered at 200 Hz, digitized at 200 Hz with a Digidata 1440 data acquisition system, and captured using pClamp 10 software (Molecular Devices, version10.7, San Jose, CA, USA).

### 5.5. Xenopus laevis Ion Channel Expression and Two-Electrode Voltage-Clamp Experiments

CsTx-13 was tested on a panel of 12 voltage-gated K_V_ channels, 5 Na_V_ channels, and 1 Ca_V_ channel, expressed in *Xenopus laevis* oocytes using the two-electrode voltage-clamp technique. We followed the protocols described in detail previously [32,33]. For the expression of Na_V_ channels (mammalian rNa_V_1.2, rNa_V_1.4, hNa_V_1.5, mNa_V_1.6, the insect channel DmNa_V_1 from *Drosophila melanogaster*, and the auxiliary subunits rb1, hb1 and TipE), K_V_ channels (mammalian rK_V_1.1, rK_V_1.2, hK_V_1.3, rK_V_1.4, rK_V_1.5, rK_V_1.6, rK_V_2.1, hK_V_3.1, rK_V_4.2, rK_V_4.3, the hERG, and *Drosophila* Shaker IR), and Ca_V_ channel (mammalian rCa_V_3.3) in *Xenopus laevis* oocytes, the linearized plasmids were transcribed using the T7 or SP6 mMESSAGE-mMACHINE transcription kit (Ambion, Carlsbad, CA, USA). In total, 50 nL of cRNA (1 ng/nl) were injected into oocytes, which were incubated in ND96 solution containing 96 mM NaCl, 2 mM KCl, 1.8 mM CaCl_2_, 1 mM MgCl_2_, and 5 mM HEPES (pH = 7.4), supplemented with 50 mg/L gentamycin sulfate. Recordings were performed using a Geneclamp 500 amplifier (Molecular Devices) controlled by a pClamp data acquisition system (Axon Instruments); bath solution was ND96. VGSC currents were evoked by a 100 ms (Nav) or 500 ms (K_V_ and Ca_V_) depolarization to the voltage corresponding to the maximal activation of the channels in control conditions from a holding potential of −90 mV. All data were obtained in at least 3 independent experiments.

### 5.6. Bioassays and LD_50_ Calculations

The fruit fly *Drosophila melanogaster* was used to determine the insecticidal activity of CsTx-1, CsTx-9, and CsTx-13 alone, and the combinations of CsTx-1 with CsTx-9 or CsTx-13, and CsTx-9 with CsTx-13. Briefly, 50 nL were injected intrathoracically into the flight muscles of 1- to 7-day-old female flies with glass capillaries. The pure neurotoxins and the combinations (1:1 molar ratio) were dissolved in 0.1 M ammonium acetate, pH = 6.1. As a negative control, 50 nL of buffer were injected.

Different toxin concentrations were injected into the flies: As for CsTx-1, 28 concentrations between 0.32 and 9.36 pmol/mg with 10 flies each (total N = 280; 5 negative controls with 10 flies each, total N = 50); for CsTx-9, 19 concentrations between 10.7 and 92.3 pmol/mg (N = 190; negative control N = 30), and for CsTx-13, 32 concentrations between 1 and 267.4 pmol/mg (N = 320; negative control N = 60). For the combination of CsTx-1 and CsTx-13, 21 concentrations between 0.05 and 1.15 pmol/mg (N = 210; negative control N = 40) were injected; for the combination of CsTx-1 and CsTx-9, 20 concentrations between 0.01 and 5.46 pmol/mg (N = 200; negative control N = 40); and for the combination of CsTx-13 and CsTx-9, 30 concentrations between 0.009 and 0.50 pmol/mg (N = 300; negative control N = 60) were injected. Calculations of the lethal dose (LD_50_), where 50% of the injected flies die of intoxication after 24 h, were done with GraphPad PRISM Vers. 6.07 (GraphPad Software, San Diego, CA, USA).

### 5.7. Microscale Thermophoresis

The principle of MST has been described in detail elsewhere [34]. K_D_ of the toxin was measured using the Monolith NT.115 (BLUE/RED) from Nanotemper Technologies GmbH, Munich, Germany. CsTx-1 and CsTx-9 were fluorescently labelled according to the manufacturer’s protocol using the L003 Monolith™ Protein Labelling kit RED-NHS with dye NT-647. Briefly, 20 μM of CsTx-1 or CsTx-9, dissolved in 20 mM MOPS, pH = 6.0, was incubated with NT-647 at a protein:dye ratio of 1:3 in a final volume of 200 μL. This particular pH condition forces the preference to label the N-terminal amine [35]. CsTx-13 was serially diluted from 10 µM to 0.305 nM in the presence of 0.75 µM-labelled toxin (CsTx-1 or CsTx-9). Measurements were performed at 23 °C by using 20% LED power and 20% IR-laser power using an NT.115 instrument. Data extraction was performed using Nanotemper M.O. Analysis software, v.1.2.101 and analysis was done on GraphPad Prism 5 (GraphPad Software, San Diego, CA, USA).

### 5.8. Molecular Modelling

Identification of the protein structure template was realized after using Protein Homolog/analogy Recognition Engine V2.0 called Phyre2 [14]. The best model was selected based on a highest sequence identity. The NMR structure of purotoxin-2 [B3EWH0; PDB: 2MZF/2MZG] [13] in water was selected. Alignment homology protein structure modelling was performed with the computer program Modeller v9.19 [15,36]. Fifty different structures were generated with constrain S-S and α-helix. The best model was selected based on the statistic values.

## Figures and Tables

**Table 1:** Estimation of the secondary structure of CT1-long and CT13-long by circular dicroism spectroscopy.

Secondary Structure Content (%)							
Peptides in Solution		α-helix	β-sheet	Turns	Unordered	Total	NRMSD ^3^
CT1-long ^4^	PBS ^1^	1	28	23	48	99	0.019
CT1-long ^4^	TFE ^2^	66	16	7	11	100	0.005
CT13-long	PBS ^1^	1	26	22	51	99	0.014
CT13-long	TFE ^2^	74	11	6	8	100	0.004

^1^ PBS: 5 mM sodium phosphate, 150 mM sodium fluoride, pH = 7.2; ^2^ TFE: 5 mM sodium phosphate, 150 mM sodium fluoride, pH = 7.2, 50% trifluoroethanol; ^3^ NRMSD: normalized root mean square deviation, calculated by DICHROWEB server / CDSSTR, reference set 1 [11,12]; ^4^ Data from [10].

## Supplementary Materials

The following are available online at https://www.mdpi.com/2072-6651/12/4/250/s1, Figure S1: Differential effects of 500 nM CsTx13 on Na_V_, K_V_ and Ca_V_ isoforms expressed in *Xenopus laevis* oocytes, Figure S2: Cytolytic effect of the C-terminal domain of CsTx-1 and CsTx-13, Figure S3: Neurotoxins amino acid sequence alignment used for 3D modelling.

## References

[1] World Spider Catalog. Version 19.5. http://wsc.nmbe.ch.

[2] Nentwig W. (2013). Spider Ecophysiology.

[3] Windley M.J., Herzig V., Dziemborowicz S.A., Hardy M.C., King G.F., Nicholson G.M. (2012). Spider-venom peptides as bioinsecticides. Toxins.

[4] Chassagnon I., McCarthy C.A., Chin Y.K.Y., Pineda S.S., Keramidas A., Mobli M., Pham V., De Silva T., Lynch J.W., Widdop R. (2017). Potent neuroprotection after stroke afforded by a double-knot spider-venom peptide that inhibits acid-sensing ion channel 1a. Proc. Natl. Acad. Sci. USA.

[5] Kuhn-Nentwig L., Stöcklin R., Nentwig W. (2011). Venom composition and strategies in spiders. Adv. Insect Physiol..

[6] Kuhn-Nentwig L., Langenegger N., Heller M., Koua D., Nentwig W. (2019). The dual prey-inactivation strategy of spiders -In-depth venomic analysis of *Cupiennius salei*. Toxins.

[7] Kozlov S., Grishin E.V. (2007). The universal algorithm of maturation for secretory and excretory protein precursors. Toxicon.

[8] Langenegger N., Koua D., Schürch S., Heller M., Nentwig W., Kuhn-Nentwig L. (2017). Identification of a precursor processing protease from the spider *Cupiennius salei* essential for venom neurotoxin maturation. J. Biol. Chem..

[9] Wullschleger B., Kuhn-Nentwig L., Tromp J., Kämpfer U., Schaller J., Schürch S., Nentwig W. (2004). CSTX-13, a highly synergistically acting two-chain neurotoxic enhancer in the venom of the spider *Cupiennius salei* (Ctenidae). Proc. Natl. Acad. Sci. USA.

[10] Kuhn-Nentwig L., Fedorova I.M., Lüscher B.P., Kopp L.S., Trachsel C., Schaller J., Vu X.L., Seebeck T., Streitberger K., Nentwig W. (2012). A Venom-derived neurotoxin, CsTx-1, from the spider *Cupiennius salei* exhibits cytolytic activities. J. Biol. Chem..

[11] Whitmore L., Wallace B. (2004). DICHROWEB, an online server for protein secondary structure analyses from circular dichroism spectroscopic data. Nucleic Acids Res..

[12] Whitmore L., Wallace B. (2008). Protein secondary structure analyses from circular dichroism spectroscopy: Methods and reference databases. Biopolymers.

[13] Oparin P.B., Nadezhdin K.D., Berkut A.A., Arseniev A.S., Grishin E.V., Vassilevski A. (2016). Structure of purotoxin-2 from wolf spider: Modular design and membrane-assisted mode of action in arachnid toxins. Biochem. J..

[14] Kelley L.A., Mezulis S., Yates C.M., Wass M.N., Sternberg M.J. (2015). The Phyre2 web portal for protein modeling, prediction and analysis. Nat. Protoc..

[15] Webb B., Sali A. (2016). Comparative protein structure modeling using MODELLER. Curr. Protoc. Protein Sci..

[16] Nadezhdin K.D., Romanovskaia D., Sachkova M.Y., Oparin P.B., Kovalchuk S.I., Grishin E.V., Arseniev A.S., Vassilevski A. (2017). Modular toxin from the lynx spider *Oxyopes takobius*: Structure of spiderine domains in solution and membrane-mimicking environment. Protein Sci..

[17] Vassilevski A., Sachkova M.Y., Ignatova A.A., Kozlov S., Feofanov A.V., Grishin E.V. (2013). Spider toxins comprising disulfide-rich and linear amphipathic domains: A new class of molecules identified in the lynx spider *Oxyopes takobius*. FEBS J..

[18] Eisenberg D. (1984). Three-dimensional structure of membrane and surface proteins. Ann. Rev. Biochem..

[19] Lee T.-H., Hall K., Aguilar M.-I. (2015). Antimicrobial peptide structure and mechanism of action: A focus on the role of membrane structure. Curr. Top. Med. Chem..

[20] Pukala T.L., Boland M.P., Gehman J.D., Kuhn-Nentwig L., Separovic F., Bowie J.H. (2007). Solution structure and interaction of cupiennin 1a, a spider venom peptide, with phospholipid bilayers. Biochemistry.

[21] Kuhn-Nentwig L., Müller J., Schaller J., Walz A., Dathe M., Nentwig W. (2002). Cupiennin 1, a new family of highly basic antimicrobial peptides in the venom of the spider *Cupiennius salei* (Ctenidae). J. Biol. Chem..

[22] Dubovskii P.V., Vassilevski A., Kozlov S., Feofanov A., Grishin E.V., Efremov R.G. (2015). Latarcins: Versatile spider venom peptides. Cell. Mol. Life Sci..

[23] Kuhn-Nentwig L. (2003). Antimicrobial and cytolytic peptides of venomous arthropods. Cell. Mol. Life Sci..

[24] Penney D., Penney D. (2013). Palaeontology: Interpretation and application of the spider fossil record. Spider Research in the 21st Century: Trends & Perspectives.

[25] Vassilevski A., Fedorova I.M., Maleeva E.E., Korolkova Y.V., Efimova S.S., Samsonova O.V., Schagina L.V., Feofanov A.V., Magazanik L., Grishin E.V. (2010). Novel class of spider toxin: Active principle from the yellow sac spider *Cheiracanthium punctorium* venom is a unique two-domain polypeptide. J. Biol. Chem..

[26] Vassilevski A., Kozlov S., Samsonova O.V., Egorova N.S., Karpunin D.V., Pluzhnikov K.A., Feofanov A., Grishin E.V. (2008). Cyto-insectotoxins, a novel class of cytolytic and insecticidal peptides from spider venom. Biochem. J..

[27] Trachsel C., Siegemund D., Kämpfer U., Kopp L.S., Bühr C., Grossmann J., Lüthi C., Cunningham M., Nentwig W., Kuhn-Nentwig L. (2012). Multicomponent venom of the spider *Cupiennius salei*: A bioanalytical investigation applying different strategies. FEBS J..

[28] Kuhn-Nentwig L., Schaller J., Nentwig W. (1994). Purification of toxic peptides and the amino acid sequence of CSTX-1 from the multicomponent venom of *Cupiennius salei* (Araneae:Ctenidae). Toxicon.

[29] Wullschleger B., Nentwig W., Kuhn-Nentwig L. (2005). Spider venom: Enhancement of venom efficacy mediated by different synergistic strategies in *Cupiennius salei*. J. Exp. Biol..

[30] Sreerama N., Woody R.W. (2000). Estimation of protein secondary structure from circular dichroism spectra: Comparison of CONTIN, SELCON, and CDSSTR methods with an expanded reference set. Anal. Biochem..

[31] Sigel E. (1987). Properties of single sodium channels translated by *Xenopus* oocytes after injection with messenger ribonucleic acid. J. Physiol..

[32] Peigneur S., Cheneval O., Maiti M., Leipold E., Heinemann S.H., Lescrinier E., Herdewijn P., De Lima M.E., Craik D.J., Schroeder C.I. (2018). Where cone snails and spiders meet: Design of small cyclic sodium-channel inhibitors. FASEB J..

[33] Sachkova M.Y., Singer S.A., Macrander J., Reitzel A.M., Peigneur S., Tytgat J., Moran Y. (2019). The birth and death of toxins with distinct functions: A case study in the sea Anemone *Nematostella*. Mol. Biol. Evol..

[34] Jerabek-Willemsen M., André T., Wanner R., Roth H.M., Duhr S., Baaske P., Breitsprecher D. (2014). MicroScale Thermophoresis: Interaction analysis and beyond. J. Mol. Struct..

[35] Sélo I., Négroni L., Créminon C., Grassi J., Wal J. (1996). Preferential labeling of α-amino N-terminal groups in peptides by biotin: Application to the detection of specific anti-peptide antibodies by enzyme immunoassays. J. Immunol. Methods.

[36] Webb B., Sali A. (2016). Comparative protein structure modeling using MODELLER. Curr. Protoc. Bioinform..

