# Supplementary material for: Neurotoxin Merging: A Strategy Deployed by the Venom of the Spider Cupiennius salei to Potentiate Toxicity on Insects
Source: Toxins (Basel). 2020 Apr 12;12(4):250. doi: 10.3390/toxins12040250 (PMC7232441; doi:10.3390/toxins12040250)
Supplement: Supplementary file 1 [file toxins-12-00250-s001.pdf]

Supplementary Materials: Neurotoxin Merging: A Strategy Deployed by the Venom of the Spider Cupiennius salei to Potentiate Toxicity on Insects

Benjamin Clémençon, Lucia Kuhn-Nentwig, Nicolas Langenegger, Lukas Kopp, Steve Peigneur, Jan Tytgat, Wolfgang Nentwig and Benjamin P. Lüscher

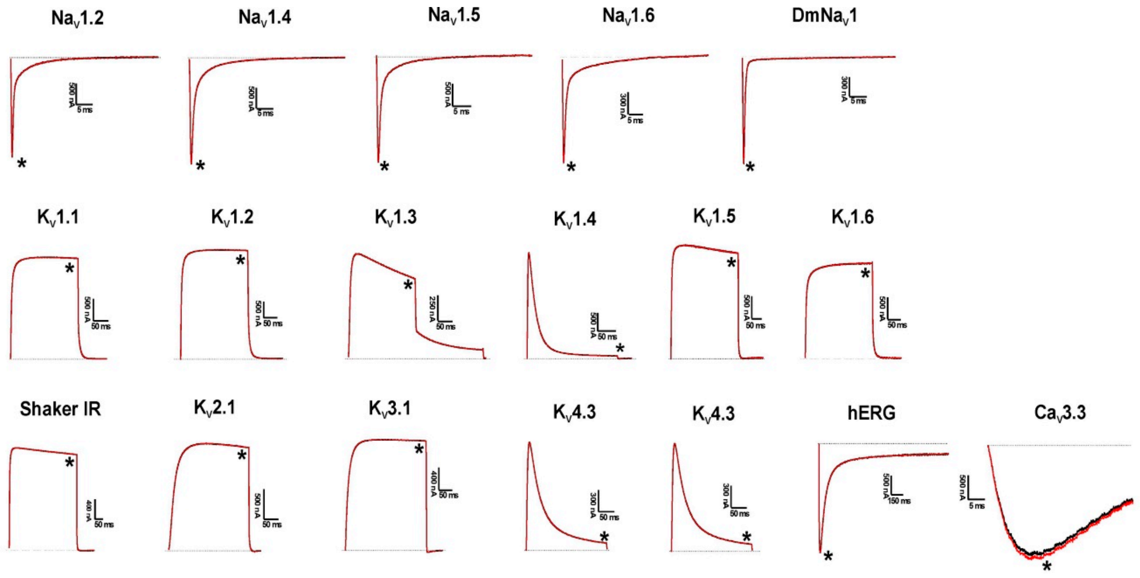

Figure S1. Differential effects of 500 nM CsTx13 on Na_V, K_V and Ca_V isoforms expressed in *Xenopus laevis* oocytes. Representative whole cell ion current traces of oocytes expressing cloned Na_V, K_V or Ca_V isoforms are shown. The dotted line indicates the zero-current level. The * indicates the steady-state current peak amplitude in the presence of 500 nM CsTx-13.

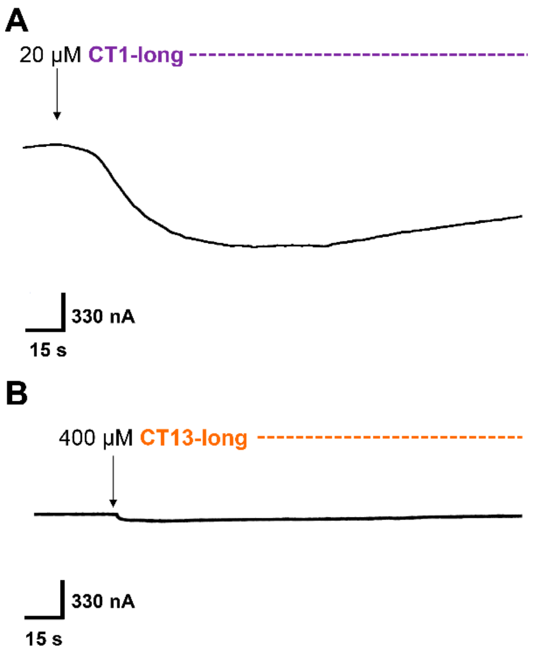

Figure S2. Cytolytic effect of the C-terminal domain of CsTx-1 and CsTx-13. (**A**) CT1-long (purple) induces a current at 20 µM concentration. The maximal current amplitude is comparable to the current amplitude induced by full length CsTx-1 (Fig. 2A). (**B**) CT13-long (orange) induced a small current at 400 µM concentration exhibiting a current amplitude of 60 nA.

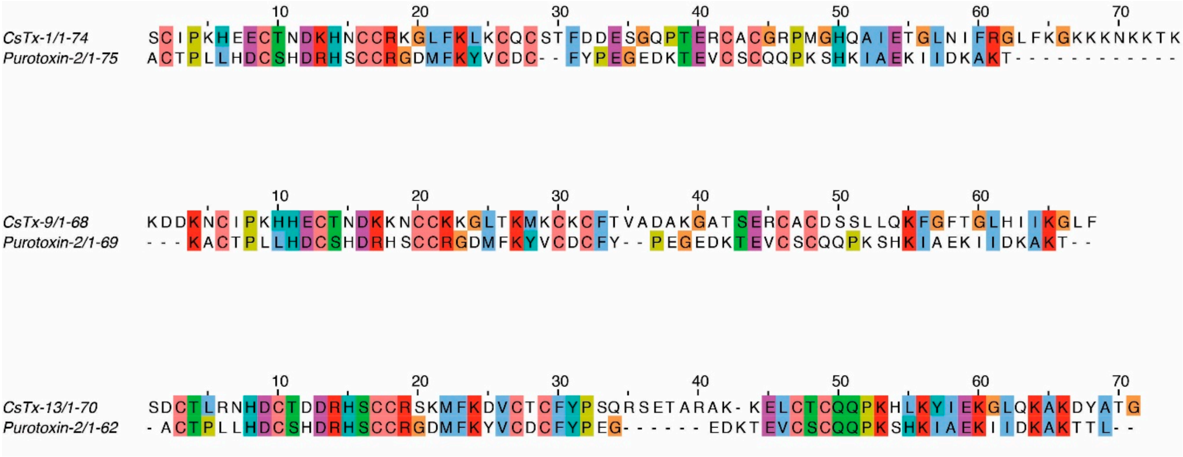

Figure S3. Neurotoxins amino acid sequence alignment used for 3D modelling. The three sequences were aligned using ClustalX program: CsTx-1, CsTx-9 and CsTx-13 to purotoxin-2. The alignment was visualized using JALVIEW program where similar as well as identical amino acids were highlight according the default JALVIEW colour code.
